# Multigeneration Sublethal Chlorantraniliprole Treatment Disrupts Nutritional Metabolism and Inhibits Growth, Development, and Reproduction of *Phthorimaea absoluta*

**DOI:** 10.3390/insects16050524

**Published:** 2025-05-15

**Authors:** Lun Li, Zunzun Jia, Kaiyun Fu, Xinhua Ding, Weihua Jiang, Xiaowu Wang, Tursun. Ahmat, Jiahe Wu, Yutong Wen, Xiaoqin Ye, Wenchao Guo, Hongying Hu

**Affiliations:** 1 College of Life Science and Technology, Xinjiang University/Xinjiang Key Laboratory of Biological Resources and Genetic Engineering, Urumqi 830017, China; lilun096@163.com (L.L.); yukio275@163.com (Y.W.); 2 Institute of Plant Protection, Xinjiang Uygur Autonomous Region Academy of Agricultural Sciences/Key Laboratory of Integrated Pest Management on Crops in Northwestern Oasis, Ministry of Agriculture and Rural Affairs/Xinjiang Key Laboratory of Agricultural Biosafety, Urumqi 830091, China; jiazunzun@163.com (Z.J.); fukaiyun000@foxmail.com (K.F.); dingxinhua1984@163.com (X.D.); wxw303528@163.com (X.W.); tu1015@163.com (T.A.); yxq13201236435@163.com (X.Y.); 3 College of Plant Protection, Nanjing Agricultural University/State Key Laboratory of Agricultural and Forestry Biosecurity, Nanjing 211800, China; jwh@njau.edu.cn; 4 Institute of Microbiology, Chinese Academy of Sciences/State Key Laboratory of Plant Genomics, Beijing 100101, China; wujiahe@im.ac.cn

**Keywords:** *Phthorimaea absoluta*, chlorantraniliprole, multigeneration, two-sex life table, larval nutrient reserves

## Abstract

*Phthorimaea absoluta*, an important pest of tomato crops, has reportedly developed high levels of resistance to the insecticide chlorantraniliprole, which has a unique mode of action and high efficacy. This study evaluated the sustained multigenerational effects of chlorantraniliprole on *P. absoluta*, focusing on resistance development, growth, development, reproductive capacity, population parameters, and nutritional indicators. After continuous selection with sublethal chlorantraniliprole for eight generations (CX-Sub_8_), bioassays showed that CX-Sub_8_ had 225.37-fold higher resistance than the susceptible strain. The age-stage, two-sex life table analysis revealed that the preadult development time and mean generation time were significantly prolonged, while population reproduction and pupal weight were reduced. Moreover, the relative fitness of CX-Sub_8_ was 0.62, and changes in the life table parameters correlated with an increase in the serial number of selection cycles. The second-instar larvae of CX-Sub_8_ presented lower triglyceride, glycerol, trehalose, free fatty acid, and protein contents than the unselected strain (CX-S_8_). Transcriptome analysis identified 2517 differentially expressed genes, with most being enriched in nutrient metabolism-related pathways, such as amino acid biosynthesis and fatty acid degradation metabolism. These results indicate that multigenerational sublethal chlorantraniliprole treatment disrupts the nutritional metabolism, and inhibits the growth, development, and reproduction of *P. absoluta*.

## Simple Summary

*Phthorimaea absoluta* (Meyrick) (Lepidoptera: Gelechiidae) is an invasive and destructive pest that significantly threatens global tomato production. Although *P. absoluta* is known to be resistant to chlorantraniliprole, the long-term effects of this insecticide on multiple generations of the pest, particularly in terms of its development and biochemical parameters, remain poorly understood. This study evaluates the sustained multigenerational effects of chlorantraniliprole on *P. absoluta*, with a focus on resistance development, life cycle parameters, and changes in nutrient reserves. Our results indicate that the resistance of *P. absoluta* significantly increased after eight consecutive generations of selection with chlorantraniliprole. The life cycle analysis revealed prolonged developmental times. Additionally, fecundity was reduced. Biochemical parameter analysis in the second-instar larvae showed significant reductions in nutrient reserves. Transcriptome analysis revealed changes in nutritional metabolism related to gene expression and pathways.

## 1. Introduction

The tomato leafminer, *Phthorimaea absoluta* (Meyrick) (Lepidoptera: Gelechiidae), is an invasive and destructive pest that threatens the global tomato industry [1]. *Phthorimaea absoluta* was originally restricted to South America [2]; however, since 2006, it has been introduced to 110 countries and territories throughout Europe, Africa, and Asia [3,4]. In China, *P. absoluta* was first reported in the Xinjiang Uygur Autonomous Region in August 2017, and then spread to 13 provinces [5]. According to @RISK model estimates, the economic loss of the tomato industry may range from 80 to 400 billion in China if no preventive measures are taken [6]. Since the larvae feed on the mesophyll tissues of tomato leaves, leaving the epidermis intact, the control efficacy of insecticides is significantly reduced [7,8]. Studies have shown that *P. absoluta* can complete 10–12 generations in a conventional tomato crop season [9]. To minimize yield losses, farmers frequently apply excessive amounts of insecticides, which has led to the development of serious resistance in *P. absoluta* to various types of insecticides, including diamides [10,11,12,13].

Over the past decade, diamide insecticides have become crucial in global agriculture, but their heavy use against *P. absoluta* has reduced their field efficacy, highlighting the need to understand resistance mechanisms [14]. Chlorantraniliprole is a diamide insecticide with a unique mode of action on the targeted ryanodine receptor, leading to uncontrolled calcium release, muscle paralysis, and death [15,16]. The high-frequency and large-area use of chlorantraniliprole imposes high and continuous selection pressure on *P. absoluta*, which may be responsible for the rapid evolution of substantial resistance [11,13,17]. The first report of chlorantraniliprole resistance in *P. absoluta* was recorded in a field-collected population from Europe [18], and so far, the resistance has evolved in Brazil, Italy, Israel, and Pakistan with a resistance ratio of 2 to 22,573-fold [12,16,19]. In China, resistance of *P. absoluta* to chlorantraniliprole has been identified in the field populations of 13 regions, with the highest resistance reaching 76.9-fold [14].

Insecticide resistance often involves enhanced metabolic activity associated with detoxification [20]. Previous studies have shown that under short-term environmental stress, insects strategically partition energy between detoxification and reproduction to maximize fitness and sustain population growth [21,22]. However, in most cases, since detoxification metabolism needs a large amount of energy, prioritizing the consumption of energy for detoxification usually leads to growth and development inhibition in insects [23]. Nutrients used by insects, including triglycerides, glycerol, trehalose, free fatty acids, proteins, and amino acids, play an important role in this process [24]. Sublethal deltamethrin and bistrifluron can increase the total protein, lipid, and carbohydrate content in *Corcyra cephalonica* and *Spodoptera exigua*, respectively [25,26]. In both cases, the nutrient reserves are significantly higher in later generations compared to earlier generations, with larval growth and development delayed. In contrast, it has also been proposed that different stress factors can reduce the total protein content in the hemolymph of silkworms, which may be due to its decomposition being caused by chemical stress [27,28]. In addition, when *Anopheles mosquitoes* are exposed to sublethal DDT for multiple generations, they have fewer lipids, sugars, and energetic reserves than sensitive ones [29]. In the field, insecticides are used repeatedly over long periods [12]. Studying pest nutrient reserve changes after multigenerational sublethal exposure helps us understand insect nutritional metabolism, growth, and development under long-term insecticide stress. However, there are still a lack of reports on *P. absoluta* in terms of the above aspects.

Insecticide resistance usually develops at the cost of a decrease in reproductive ability and relative fitness [30]. Recent reports have shown that sublethal concentrations of insecticides can inhibit insect reproduction and decrease fitness. For example, the resistance of *Plutella xylostella* and *Musca domestica* to chlorantraniliprole and *Spodoptera exigua* to deltamethrin lead to extended development, decreased survival rates, and decreased fecundity [31,32,33]. In contrast, some studies have also shown that target insects enhance their reproductive ability and fitness after exposure to insecticides. For example, when *Nilaparvata lugens* and *Sogatella furcifera* are exposed to sublethal concentrations of nitenpyram and triazophos, respectively, their fecundities are significantly increased [34,35]. *Phthorimaea absoluta* has multiple generations in a year [9]. Despite the known resistance of *P. absoluta* to chlorantraniliprole, the effects of this insecticide on the life history traits, relative fitness, and nutrient metabolism of the pest across multiple generations remain poorly understood still.

Here, we attempt to verify the hypothesis that multigenerational treatment with sublethal chlorantraniliprole will disrupt nutritional metabolism and simultaneously inhibit the growth, development, and reproduction of *P. absoluta*. This is achieved through an in-depth examination of four key aspects. First, we assess the evolution of resistance in *P. absoluta* following sublethal chlorantraniliprole treatment during the second larval stage across multiple generations. Second, we investigate the resulting growth, development, and reproduction of the pest. Third, we analyze the physiological features of the second-instar larvae, with a focus on the nutrient reserves. Fourth, we analyze the expression levels of differentially expressed genes associated with nutritional metabolism in the transcriptome.

## 2. Materials and Methods

### 2.1. Insects and Chemicals

The *P. absoluta* susceptible strain (SS) used for establishing the susceptibility baseline in this study was collected in 2020 from Aksu Prefecture (41.09° N, 80.24° E), Xinjiang, and maintained for >20 generations without exposure to insecticides. More than 1000 fourth-instar larvae of a resistant population of *P. absoluta* (CX, F_0_ generation) were collected from Tsabchal Xibe Autonomous County (43.87° N, 81.26° E), lli Kazakh Autonomous Prefecture, Xinjiang Uygur Autonomous Region, in 2022. All test insects (SS and CX) were reared on fresh tomato plants (Tian Fen 2, only in a vegetative state) in the laboratory in insect-rearing cages (0.75 × 0.75 × 0.75 m) at 25 ± 1 °C, with a relative humidity of 30 ± 5%, and with a 16 L:8 D photoperiod.

Chlorantraniliprole (purity ≥ 99%; Shanghai Macklin Biochemical Technology Co., Ltd., Shanghai, China), dimethyl sulfoxide (DMSO; Tianjin Beilian Fine Chemicals Development Co., Ltd., Tianjin, China), and Triton X-100 (Beijing Solarbio Science Technology Co., Ltd., Beijing, China) were used.

### 2.2. Bioassays

The whole-leaf dip method was used to determine susceptibility to chlorantraniliprole in submerged tomato leaves according to the Insecticide Resistance Action Committee method No. 022 (https://irac-online.org/methods/tuta-absoluta-larvae/, accessed on 7 October 2023) [10,17]. Accurate dilutions at 500, 125, 50, 12.5, 3.125, and 0.78125 mg·L^−1^ were prepared with a 0.1% Triton X-100 aqueous solution control containing DMSO. Briefly, round tomato leaf discs with a diameter of 4–5 cm or entire leaves were immersed in serial insecticide concentrations for 20 s. Treated leaves were allowed to dry for 0.5–1 h at room temperature (25 ± 1 °C) and then inserted into pre-solidified six-well plates containing 1% agar. Five second-instar larvae were placed in each well, and the edges of the six-well plates were sealed with a sealing film. All bioassays were incubated (25 ± 1 °C, 30 ± 5% relative humidity, 16 L:8 D photoperiod) for 48 h to assess larval survival. The larvae were considered dead if they did not respond or were severely deformed by touching the body with the tip of a brush. Each concentration treatment was repeated six times.

### 2.3. Selection Experiments

In the selection experiments, the concentration of chlorantraniliprole used to select each subsequent generation was LC_25_, based on the bioassay results from the previous generation. The larvae of the CX population were divided into two sub-populations during the F_1_ generation. One sub-population was selected for eight consecutive generations by exposing second-instar larvae to tomato leaves treated with LC_25_ chlorantraniliprole and was named the CX-Sub strain. Strain CX-Sub-n represented the strain selected for *n* generations consecutively. The second sub-population of larvae was the CX-S strain, and it was fed the solvent-treated tomato leaves without exposure to insecticides (eight generations were unselected and named CX-S_1_ to CX-S_8_, with CX-S_8_ acting as the control strain). The CX-S strain was reared in the laboratory in parallel with the CX-Sub strain. Resistance ratio (RR) values were estimated as the LC_50_ of the resistant strain divided by the LC_50_ of the susceptible strain (SS).

### 2.4. Life Table Data, Study, and Analysis

An age-stage, two-sex life table was constructed using the CX-Sub_2_, CX-Sub_4_, CX-Sub_8_, and control strains. First, 150 eggs were randomly collected within 12 h of the peak laying period of female adults, and hatched under the same insect-rearing conditions as described above. Next, 100 newly hatched larvae were randomly selected, placed in Petri dishes (9 cm diameter, one individual/Petri dish, No. 1–100), and provided with fresh tomato leaves. The egg incubation and larval development times were recorded every day until pupation. The development times of all life stages were recorded. Thirty randomly selected pupae were weighed and placed in individual Petri dishes until adult emergence, and male and female individuals that emerged on the same day were paired and allowed to lay eggs. Finally, the duration of the pupal stage, longevity, survival, and oviposition of *P. absoluta* were recorded daily until the adult died. We used the age-stage, two-sex life table theory, and the Twosex-MSChart program to analyze the life table raw data [36,37]. The basic life table parameters included the age-stage survival rate (*S_xj_*) (where *x* is the age and *j* is the stage), age-specific survival rate (*l_x_*), female age-specific fecundity (*f_xj_*), age-specific fecundity (*m_x_*), age-stage life expectancy (*e_xj_*), and age-stage reproductive value (*v_xj_*). The *s_xj_*, *l_x_*, *m_x_*, *e_xj_*, and *v_xj_* values were calculated using Equations (1)–(5):

(1) $$s_{xj}=\frac{n_{xj}}{n_{01}}$$

(2) $$l_{x}=\sum_{j=1}^{\beta }s_{xj}$$

(3) $$m_{x}=\frac{\sum_{j=1}^{\beta }s_{xj}f_{xj}}{\sum_{j=1}^{\beta }s_{xj}}$$

(4) $$e_{xj}=\sum_{i=x}^{\infty }\sum_{y=j}^{\beta }{s’}_{iy}$$

(5) $$v_{xj}=\frac{e^{r(x+1)}}{s_{xj}}\sum_{i=x}^{\infty }e^{-r(i+1)}\sum_{y=j}^{\beta }{s’}_{iy}f_{iy}$$

where *n*_01_ is the number of eggs used at the beginning of the life table study, *n_xj_* is the number of individuals surviving to age *x* and stage *j*, and *β* is the number of stages. The *s’_iy_* represents the probability that the individual survives to age *i* and stage *y*. The *f_iy_* represents the number of eggs laid by an individual of age *i* and stage *y* [37].

The net reproductive rate (*R*_0_), intrinsic rate of increase (*r*), finite rate of increase (*λ*), and mean generation time (*T*) are important parameters for describing population characteristics and were calculated using Equations (6)–(9):

(6) $$R_{0}=\sum_{x=0}^{\infty }l_{x}m_{x}$$

(7) $$\sum_{x=0}^{\infty }e^{-r(x+1)}l_{x}m_{x}=1$$

(8) $$\lambda =e^{r}$$

(9) $$T=\frac{\ln R_{0}}{r}$$

Life table parameters were mapped using Origin 2021 software (Northampton, MA, USA). The mean value and standard error of the life table parameters were calculated using the bootstrap method with 100,000 replications [38]. The significance of the differences between the parameters was calculated using a paired bootstrap test program.

The relative fitness (*Rf*) was analyzed using the method outlined by Abbas et al. [39] and calculated as *Rf* = *R*_0_ of the CX-Sub strain/*R*_0_ of the control strain.

Pearson’s correlation analysis was employed to analyze the correlations between the life table parameters and *P. absoluta* resistance selection across generations. All data were obtained using the bootstrap method with 100,000 replications, including the life table parameters of the CX-Sub_2_, CX-Sub_4_, and CX-Sub_8_ strains. Origin 2021 software (Northampton, MA, USA) was utilized to perform the Pearson correlation analysis [40].

### 2.5. Determination of Nutrient Reserves

Healthy second-instar larvae of the same size were randomly selected from the control and CX-Sub_8_ strains. Firstly, 100 mg of numerous intact second-instar larvae were homogenized in 1000 μL of the extraction solution according to the instruction manual. Then, the nutrient reserves were determined using specific substance determination kits (Suzhou Grace Biotechnology Co., Ltd., Suzhou, China). The measured nutrients included triglyceride (G0910W), glycerol (G0912W), trehalose (G0553W96), free fatty acids (G0927W96), proteins (G0418W), and amino acids (G0415W). An Epoch-cn microplate reader (Agilent BioTek, Winooski, VT, USA) was used to determine the absorbance of the reaction fluid of the nutrients. Each assay was performed in triplicate and repeated three times. The results were calculated according to the manufacturer’s instructions.

### 2.6. Transcriptome Sequencing

Transcriptome sequencing was performed by Biomarker Technologies (Beijing, China). Total RNA was extracted from three replicate pools of 50 whole second-instar larvae of each strain (CX-Sub_8_ and Control) with TRIzol reagent (Life technologies, Carlsbad, CA, USA). The concentration and purity of the RNA were measured using a NanoDrop 2000 (Thermo Fisher Scientific, Wilmington, DE, USA). RNA integrity was assessed using the RNA Nano 6000 Assay Kit with an Agilent 2100 Bioanalyzer System (Agilent Technologies, Santa Clara, CA, USA). The cDNA libraries were sequenced on the Illumina NovaSeq 6000 platform and analyzed using the bioinformatics analysis tool on the BMK Cloud online platform (www.biocloud.net) [41]. Briefly, high-quality clean reads were generated by removing low-quality fragments and junctions from the raw reads, which were mapped to the reference genome using HISAT2 (GenBank no. GCA_027580185.1). The reads were assembled using StringTie to reconstruct the transcriptome for subsequent analyses [42]. Gene transcript levels were calculated using the fragments per kilobase of exon model per million mapped reads (FPKM) method [43]. The genes with a fold change (FC) ≥ 2 and false discovery rate (FDR) ≤ 0.01 were designated as differentially expressed genes (DEGs), which were subjected to enrichment analysis by Gene Ontology (GO) and Kyoto Encyclopedia Genes of Genomes (KEGG) annotations. Rich factor = DEG number/total gene number identified from the transcriptome of a certain process.

### 2.7. Validation of Transcriptomic Data with Quantitative Real-Time PCR (qRT-PCR)

To verify the transcriptome data, nine genes (from the top three KEGG-enriched entries: biosynthesis of amino acids, fatty acid degradation, and glycine, serine, threonine metabolism) were randomly selected from the DEG list for qRT-PCR. Total RNA was extracted from the samples in Section 2.6, following the standard protocol of the Total RNA Extraction Reagent (Vazyme, Nanjing, China). First-strand cDNA was generated from 1 μg total RNA using HiScript III RT SuperMix (Vazyme, Nanjing, China). The reaction volume of the qRT-PCR was 20 μL: RNase-free water (8.2 μL), qPCR SYBR Green Master Mix (10 μL), forward primer (0.4 μL, 10 μM), reverse primer (0.4 μL, 10 μM), and cDNA template (1 μL). The qPCR procedure was as follows: pre-denaturation at 95 °C for 30 s; then 40 cycles were conducted (denaturation at 95 °C for 10 s, annealing at 60 °C for 30 s). The stably expressed genes TaEF1α (GenBank: MZ054826) and TaRPL28 (GenBank: MZ054829) were used as the reference genes [44]. qRT-PCR was conducted using three biological and technical replicates. The relative expression levels were calculated using the Ct (2^−ΔΔCt^) method [45]. Primer sequences for all genes are presented in Table 1.

### 2.8. Statistical Analysis

Bioassays were analyzed by probit analysis via SPSS version 22.0 software (SPSS Inc., Chicago, IL, USA) [46]. The relative expression levels and nutrient reserve results were compared by Student’s *t*-test using the SPSS 22.0 software as well. All data were expressed as the mean ± standard error of the mean (SEM).

## 3. Results

### 3.1. Selection of P. absoluta Resistance to Chlorantraniliprole

CX-Sub_8_ exhibited a 225.37-fold higher resistance to chlorantraniliprole than a susceptible strain (SS; Table 2). The LC_50_ value increased from 6.741 to 6.933 mg·L^−1^ during the first two generations, with a relatively slow increase in resistance. Subsequently, the resistance level increased rapidly in the third generation, with the LC_50_ increasing to 36.5 mg·L^−1^ and reaching a high resistance level (RR = 214.73-fold). The resistance level tended to stabilize between the fourth and eighth generations. Despite experiencing upward and downward fluctuations, the overall trend demonstrated a gradual increase. The LC_50_ values of the unselected strain (CX-S) for chlorantraniliprole are shown in Table S1.

### 3.2. Inhibitory Effect of Chlorantraniliprole on the Growth, Development, and Reproduction of P. absoluta

**Figure 1:**
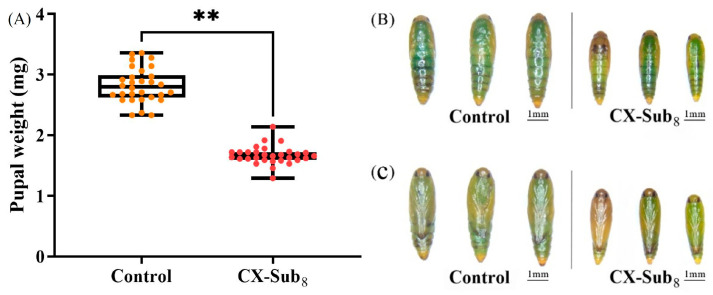
Effects of chlorantraniliprole on pupae of different strains of *P. absoluta*. (**A**) Pupal weight, (**B**) dorsal surface of control and CX-Sub_8_ pupae, (**C**) ventral surface of control and CX-Sub_8_ pupae. The pupal weight was assessed using a Student’s *t*-test. ** indicates a significantly different at the level of *p* < 0.01.

CX-Sub completed development and produced offspring. However, the biological parameters of CX-Sub differed significantly among generations (Table 3). The preadult duration of CX-Sub_4_ and CX-Sub_8_ was significantly prolonged by 0.98 and 2.45 days, respectively, compared to the control. Meanwhile, the average number of eggs per female adult in the CX-Sub_8_ was 48.56, which was 2.01-fold lower than the control, and the oviposition time was shortened by 1.66 days. The preadult time, female adult longevity, female adult ratio, adult preoviposition period (APOP), and total preoviposition period (TPOP) of CX-Sub increased gradually with the increase in the serial number of the selection cycles, while egg production was reduced. Finally, compared to the control group, the average pupal weight of CX-Sub_8_ decreased significantly by 1.15 mg (Figure 1A). Compared to the dorsal and ventral surfaces of the pupae in the control group, the pupae of CX-Sub_8_ were significantly smaller in size (Figure 1B,C).

### 3.3. Effects of Chlorantraniliprole on the Life Table Parameters of the F_2_, F_4_, and F_8_ Generations of P. absoluta

**Figure 2:**
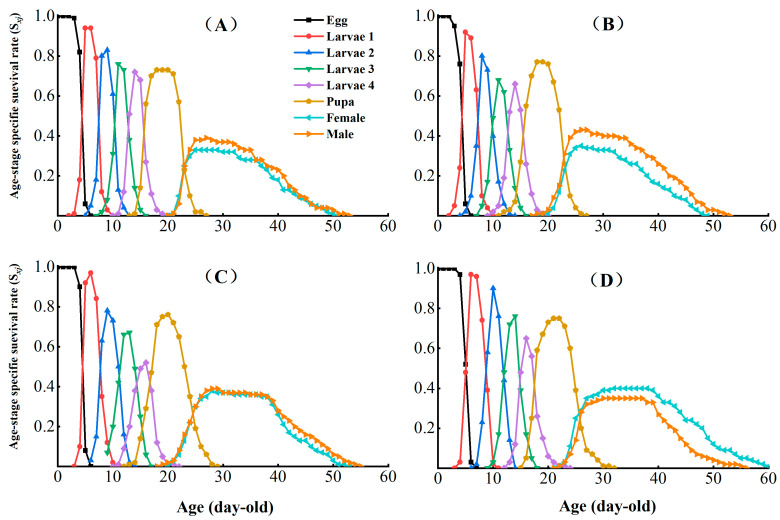
Effects of chlorantraniliprole on the age-stage survival rate (*S_xj_*) of different strains of *P. absoluta*. (**A**) Control, (**B**) CX-Sub_2_, (**C**) CX-Sub_4_, (**D**) CX-Sub_8_.

The age-stage survival (*S_xj_*) in the different CX-Sub generations assessed the probability of egg survival to age *x* and stage *j* (Figure 2). Under sublethal concentration conditions, no significant effect was observed on the survival rate of *P. absoluta* larvae with an increase in the serial number of selection cycles. However, the peak survival of adult males decreased by 0.08. In addition, the *S_xj_* curves of adult females ended later in the CX-Sub strain than the control, suggesting that LC_25_ treatment prolonged the survival of *P. absoluta* adult females.

**Figure 3:**
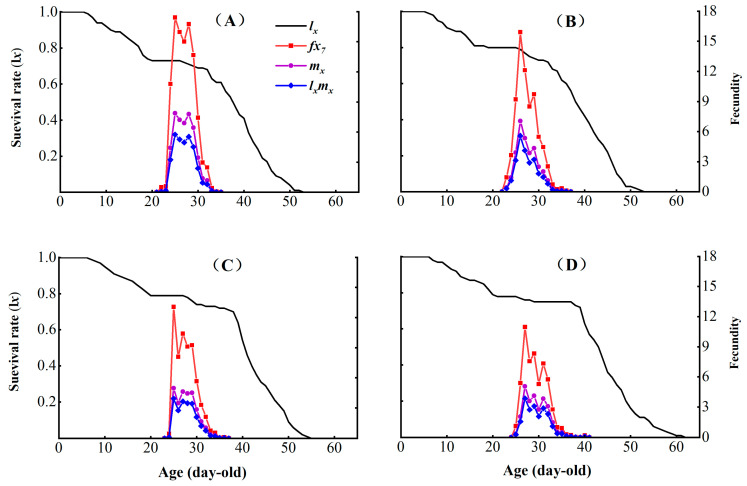
Effects of chlorantraniliprole on the age-specific survival rate (*l_x_*), female age-specific fecundity (*f_x_*), age-specific fecundity of population (*m_x_*), and age-specific net reproductive rate of the population (*l_x_ m_x_*) of different strains of *P. absoluta*. (**A**) Control, (**B**) CX-Sub_2_, (**C**) CX-Sub_4_, (**D**) CX-Sub_8_.

The control had the highest peak female age-specific fecundity value (*f_x7_*) = 17.42, occurring on day 25 (Figure 3). In contrast, CX-Sub_8_ had the lowest peak *f_x7_* = 10.97 on day 27. The control strain had the highest peak age-specific net reproductive rate of population (*l_x_m_x_*) value = 5.75 on day 25, while CX-Sub_8_ had the lowest *l_x_m_x_* peak (3.84) on day 27. Additionally, the peak age-specific fecundity of population (*m_x_*) values for the control, CX-Sub_2_, CX-Sub_4_, and CX-Sub_8_ were 7.88, 7.05, 4.96, and 5.05, respectively, aligning with the occurrence times of the peak female age-specific fecundity values of *f_x7_*.

**Figure 4:**
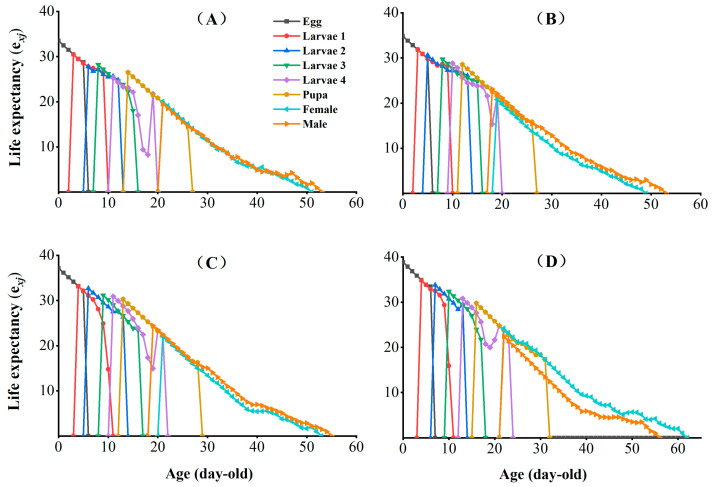
Effects of chlorantraniliprole on the life expectancy (*e_xj_*) of different strains of *P. absoluta*. (**A**) Control, (**B**) CX-Sub_2_, (**C**) CX-Sub_4_, (**D**) CX-Sub_8_.

The age-stage-specific life expectancy (*e_xj_*) indicates the expected life span of an individual of age *x* and stage *j* in the same stage after age *x* (Figure 4). The *e_xj_* of the egg, larval, pupal, and adult stages were increased in CX-Sub_2_, CX-Sub_4_, and CX-Sub_8_ compared to the control.

**Figure 5:**
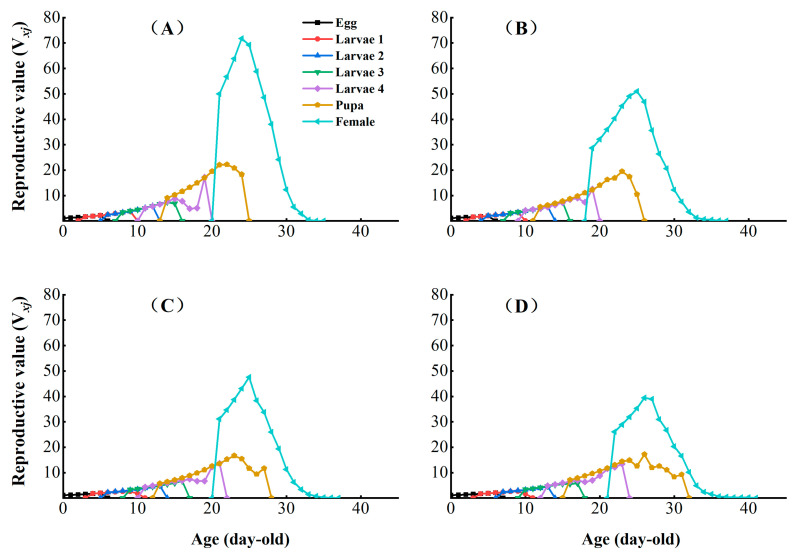
Effects of chlorantraniliprole on the age-stage reproductive value (*V_xj_*) of different strains of *P. absoluta*. (**A**) Control, (**B**) CX-Sub_2_, (**C**) CX-Sub_4_, (**D**) CX-Sub_8_.

The age-stage reproductive value (*V_xj_*) represents the individual contribution of age *x* and stage *j* to the future of the population (Figure 5). The peak reproductive values of CX-Sub_2_, CX-Sub_4_, and CX-Sub_8_ females were lower than those for the control strain. The sublethal concentration of chlorantraniliprole significantly reduced the *Vxj* of *P. absoluta* with an increase in the serial number of the selection cycles.

**Figure 6:**
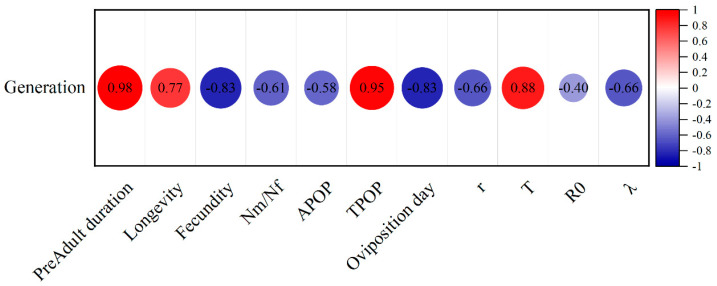
Pearson’s correlation heatmap of each life table parameter analyzed in three strains of *P. absoluta* (CX-Sub_2_, CX-Sub_4_, and CX-Sub_8_).

In CX-Sub, the preadult duration (*R* = 0.98), longevity (*R* = 0.77), TPOP (*R* = 0.95), and *T* (*R* = 0.88) were positively correlated with the serial number of the selection cycles (Figure 6). Meanwhile, the fecundity (*R* = −0.83), male-to-female ratio (*R* = −0.61), oviposition days (*R* = −0.83), *r* (R = −0.66), and *λ* (*R* = −0.66) were negatively correlated with the selection cycle.

### 3.4. Effects of Chlorantraniliprole on the Population Parameters of the F_2_, F_4_, and F_8_ Generations of P. absoluta

The *GRR*, *R*_0_, *r_i_*, and *λ* values slightly decreased in CX-Sub_2_, but significantly decreased in CX-Sub_4_ and CX-Sub_8_ compared to the control. The mean generation time (*T*) in CX-Sub_8_ was 30.17 d, which was 2.35 d longer than that in the control strain (Table 4). The relative fitness (*R_f_*) values of CX-Sub_2_, CX-Sub_4_, and CX-Sub_8_ were 0.74, 0.65, and 0.62 lower than for the control strain, respectively (Table 4). This suggests that the fitness of CX-Sub suffered a cost due to resistance.

### 3.5. Chlorantraniliprole Reduces Nutrient Reserves in the Second-Instar Larvae of P. absoluta

**Figure 7:**
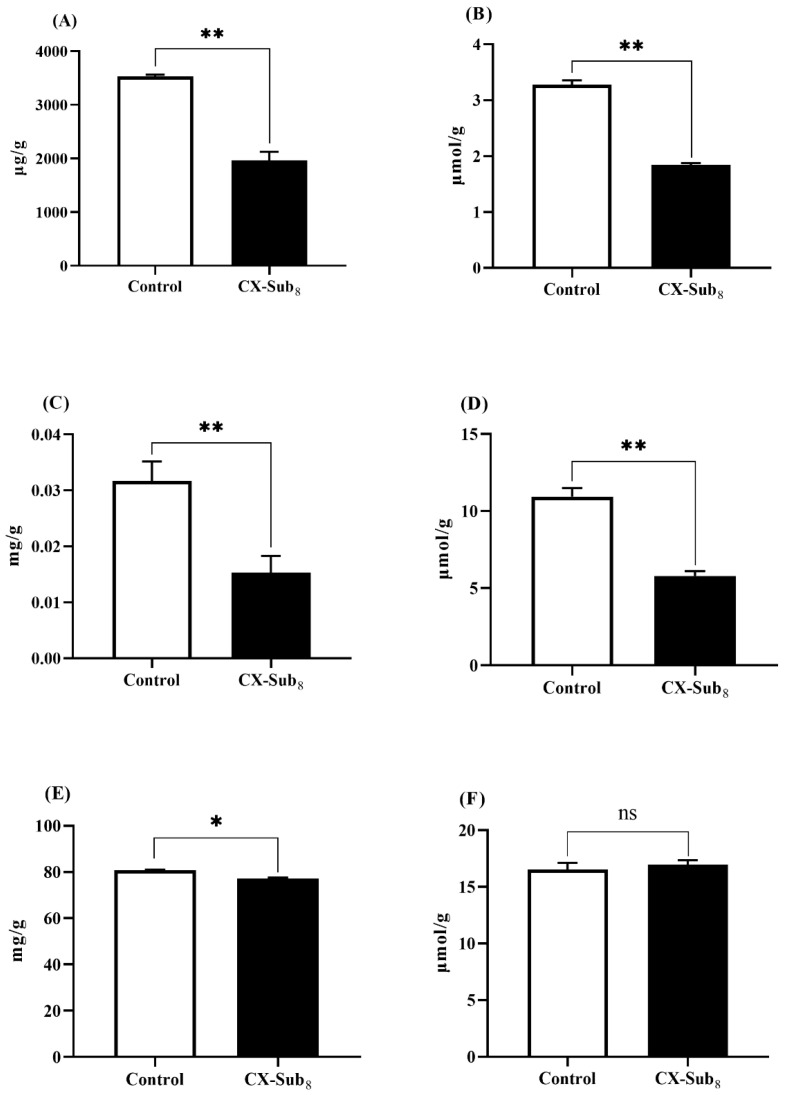
Effects of chlorantraniliprole on *P. absoluta* nutrient reserves. (**A**) Triglycerides, (**B**) glycerol, (**C**) trehalose, (**D**) free fatty acids, (**E**) proteins, and (**F**) amino acids. * indicates a significantly different at the level of *p* < 0.05, ** indicates a significantly different at the level of *p* < 0.01, and ns is not significantly different (*p* > 0.05).

Compared to the control strain, the content of triglycerides, glycerol, trehalose, free fatty acids, and proteins were significantly reduced (*p* < 0.05) in CX-Sub_8_ larvae by 44.4%, 43.9%, 53.1%, 47.1%, and 4.5%, respectively. In contrast, the amino acid content increased slightly, but not significantly (Figure 7). These data showed that after continuous selection with chlorantraniliprole for eight generations, significant changes occurred in the larval nutrient reserves of *P. absoluta*.

### 3.6. Disruption of Nutritional Metabolism in P. absoluta by Chlorantraniliprole Revealed Through Transcriptome Sequencing

**Figure 8:**
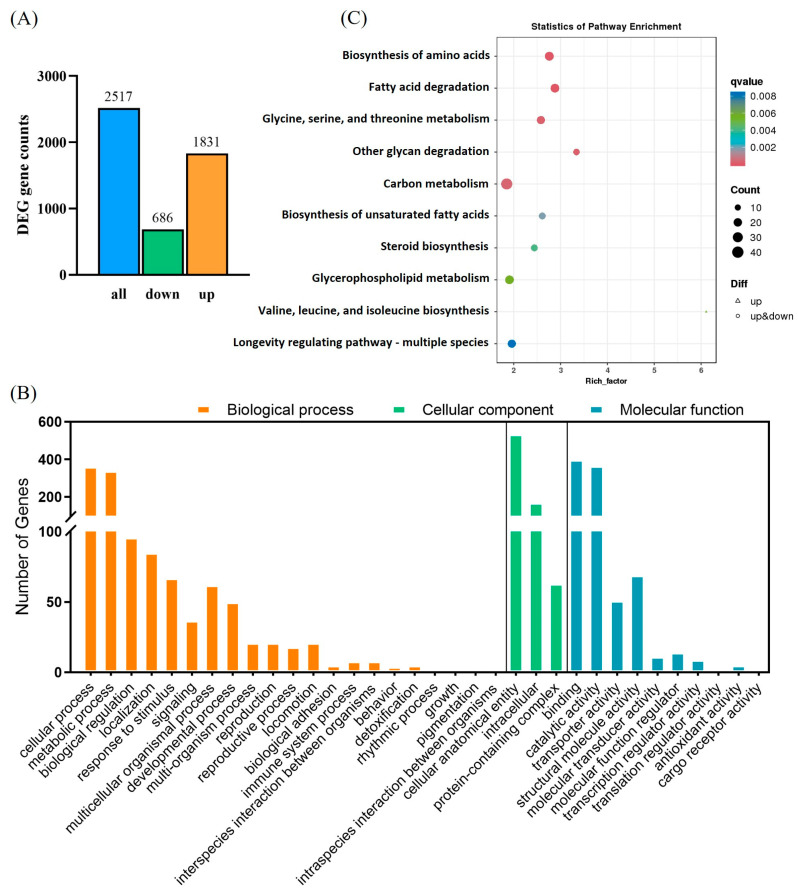
Analysis of differentially expressed genes (DEGs) between the control and CX-Sub_8_ strains of *P. absoluta*. (**A**) Number of upregulated and downregulated DEGs in the control and CX-Sub_8_ strains. (**B**) Gene Ontology (GO) analysis of DEGs in the control and CX-Sub_8_ strains. (**C**) Kyoto Encyclopedia of Genes and Genomes (KEGG) analysis of DEGs in the control and CX-Sub_8_ strains. Processes with a *q* value < 0.01 are significantly enriched.

**Figure 9:**
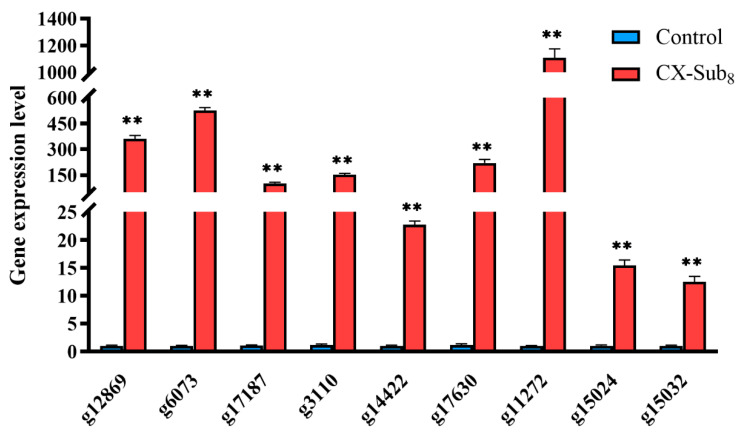
Comparison of the RNA-seq and qPCR validated expression of differentially expressed genes (DEGs) of the control and CX-Sub_8_ strains of *P. absoluta*. ** indicates a significantly different at the level of *p* < 0.01.

Sequencing generated 38.11 Gb of clean data with an average of 6.18 Gb per data library. The proportion of Q30 bases was >93.61%. The alignment rate to the reference sequence was 79.60–82.64%. A total of 2517 DEGs were detected, with 1831 upregulated and 686 downregulated, in CX-Sub_8_ versus the control (Figure 8A). GO annotation analysis showed that the primary biological processes associated with the DEGs were cellular, metabolic, and biological. DEGs were enriched in cellular components related to cellular anatomical entities, as well as intracellular and protein-containing complexes. Under molecular functions, binding, catalytic activity, and structural molecule activity were associated with most of the DEGs (Figure 8B). In addition, KEGG pathway enrichment analysis revealed that the DEGs were enriched in the biosynthesis of amino acids; fatty acid degradation; glycine, serine, and threonine metabolism; other glycan degradation; carbon metabolism; and the biosynthesis of unsaturated fatty acids (Figure 8C, Table S2). qRT-PCR was used to verify the transcriptome data by analyzing the expression levels of nine randomly selected upregulated genes (Table 1). The variations in gene expression levels were consistent with the transcriptome data (*p* < 0.05), demonstrating the reliability of the RNA-seq results (Figure 9).

## 4. Discussion

In this study, the resistance level of CX-Sub to chlorantraniliprole increased from medium to high levels through three generations (RR = 214.73-fold). This is consistent with the results of Silva et al. [47] and Jallow et al. [48], who reported that resistance to chlorantraniliprole in *P. absoluta* developed rapidly after six to eight generations of selection in Kuwait and the United States, respectively. The field populations of *P. absoluta* in Italy developed high levels of resistance to chlorantraniliprole (RR = 742-fold) after only four generations [15]. Similar results were reported for *P. xylostella* in China and the United Kingdom [49,50].

When a pest is resistant to a pesticide, identifying the resistance mechanisms and suppressing the resistance in the field are key to pest management [51]. A fitness cost is typically required for insects to develop resistance [30]. In the current study, CX-Sub showed a gradual decrease in *R*_0_ and *GRR* with an increase in the serial number of the selection cycles, which is consistent with the significant decrease in average fecundity, *R*_0_, *r_i_,* and *λ* in an *S. exigua* chlorantraniliprole-resistant strain [52]. However, unlike *P. xylostella* Sub strains (selected by LC_25_ spinosad for multiple generations), despite its higher resistance, the sublethal effect on the Sub strains decreased as the serial number of the selection cycles increased. Compared to the susceptible strain, the Sub-5 strains showed no differences in *r_m_*, *R*_0_, *λ*, and *GRR.* Notably, the Sub-10 strain had higher fecundity and shorter larval development. Thus, multigeneration spinosad selection imposed no corresponding energy or fitness costs on *P. xylostella* [53]. In this study, the effect of sublethal chlorantraniliprole on the *P. absoluta* Sub strains did not decrease as selection cycle serial numbers increased. One possible explanation is that chlorantraniliprole disrupts the normal neural tissues of insects [54], leading to motor disorders and feeding disruption [55]. This may cause larvae to spend more time feeding on leaves, compensating for their low energy reserves by consuming more food, resulting in extended preadult periods and decreased fecundity [56]. In addition, after exposure to insecticides, the balance between detoxification and development is disrupted [57]. Some of the insect’s energy is directed toward the metabolism of detoxification, reducing the energy essential for insect development and reproduction [24].

Generally, fecundity reflects the development of insect populations [58]. Previous studies have found that after *P. absoluta* and *H. armigera* are exposed to tetraniliprole and chlorantraniliprole, respectively, the female adults’ fecundity will be significantly reduced [59,60]. Conversely, these findings suggest that exposure to low doses of a pesticide might increase the fecundity of insects, but rising fecundity and offspring population could be inhibited by the LC_50_ of insecticides [61]. For example, *Tetranychus urticae* exposed to the LC_10_ of chlorfenapyr had increased fecundity and longer oviposition days, but these traits decreased at higher concentrations [62]. Similarly, an increase in the fecundity of second-generation *Daphnia carinata* was observed at low concentrations of chlorpyrifos [63]. Our results showed that multigeneration LC_25_ chlorantraniliprole application significantly reduced the fecundity of CX-Sub compared to the control. The decrease in female offspring production might be attributable to the following factors: First, the relatively high sublethal concentration of chlorantraniliprole [64]. Second, insecticide exposure damages most viable oocytes [65]. Third, chlorantraniliprole significantly disrupts insect mating behavior [66]. Moreover, the pupal weight of *P. absoluta*, which is closely related to egg production, enables females with higher pupal weights to lay more eggs for the next generation [67]. This positive correlation has also been reported in *P. xylostella* [68], *H. armigera* [69,70], and other species [71]. However, the reasons for the reduced fecundity of *P. absoluta* treated through more generations with sublethal chlorantraniliprole require further investigation.

When target insects are exposed to pesticides during the preadult stage, the survival rates of the sexes differ [13]. Although sexually reproducing species typically have a sex ratio of 1:1, several mechanisms may cause this ratio to deviate, such as when sex-asymmetric inbreeding occurs or through exposure to the insecticide [72,73]. The female ratio of CX-Sub increased gradually with the serial number of the selection cycles, exhibiting a significant positive correlation. In contrast, treating *P. absoluta* with indoxacarb, carbendazim, and abamectin increased the male ratio [74]. Differences in the sex ratio might result from insecticide exposure affecting the mating frequencies and conception rates of male and female insects [75] or causing significant differences in insecticide susceptibility due to locomotory and physiological responses [76].

Previous findings regarding insect nutritional reserves were that females exposed to insecticides yield heavier offspring with more lipid storage than non-exposed ones [77]. For instance, sublethal phosphine significantly increased protein, lipid, and carbohydrate content across generations of *C. cephalonica* [25]. Despite many insecticides reducing insect feeding efficiency, insects might boost their nutritional reserves and reduce body metabolic components to counter insecticide-induced energy stress [78]. This potentially explains the post-exposure increase in their offspring’s nutritional reserves. In contrast to the findings described above, our results showed that the multigeneration sublethal chlorantraniliprole treatment disrupted nutritional metabolism in *P. absoluta*, leading to changes in nutrient reserves. Compared to the control strain, nutrient reserves (triglyceride, glycerol, trehalose, free fatty acid, and protein content) in the CX-Sub_8_ strain were significantly decreased. Similarly, Piri et al. documented that sublethal concentrations of spinosad decreased the carbohydrate, protein, and lipid content in *Glyphodes pyloalis* [79]. They suggested that this phenomenon may be due to the food rejection effect of spinosad. Food rejection may also occur in the response of *P. absoluta* to chlorantraniliprole. However, the reduced energy reserves of carbohydrates, lipids, proteins, and glycogen in *P. absoluta* also reflect the high energetic cost of the detoxification mechanism [80]. Moreover, previous research has shown that metabolic resistance to insecticides uses resources essential for development [24]. These changes in biochemical parameters also supported the KEGG analysis in finding that the amino acid biosynthesis, fatty acid degradation, and carbon metabolism significantly enriched DEGs. Significant differences in amino acid content were not observed between CX-Sub_8_ and the control. One probable reason is that under insecticidal stress, when insects face insufficient energy reserves, they consume proteins to maintain the free amino acid content in the hemolymph [27,81]. Whether this phenomenon also exists in *P. absoluta* warrants further investigation.

Numerous studies have shown that increased insecticide resistance leads to reduced relative fitness, which may be associated with nutrient reserves and metabolism [30]. Changes in the fitness of resistant insects can disrupt normal physiological functions and create an imbalance in nutrient and energy metabolism allocation [60,68]. However, a more thorough analysis from a molecular perspective may be necessary to explore the relationship between the characteristics, such as the prolonged larval development period, reduced fecundity, and decreased pupal weight in *P. absoluta* after multigenerational sublethal treatment with chlorantraniliprole and the nutritional metabolism mechanism.

## 5. Conclusions

Our results show that resistance to chlorantraniliprole developed rapidly in *P. absoluta*, with medium to high resistance levels developed in only three generations. Moreover, multigenerational treatment with chlorantraniliprole led to longer larval development, reduced fecundity and relative fitness, and significant decreases in nutrient-related biochemical parameters compared to the unselected strain. These results provide a new perspective for further analysis of the relationship between resistance and nutrient metabolism, offering a theoretical basis for the effective application of insecticides in pest management.

## Figures and Tables

**Table 1:** Primers used in the qRT-PCR synthesis.

Gene	GenBank ID	NR: Description	E Value	Per. Ident	Direction	Primer Sequences (5′-3′)
g12869	KAJ2938428.1	fatty acid desaturase domain-containing protein [*Phthorimaea operculella*]	0.0	95.43%	F	ATCGGCTGGCTCATGATGAA
					R	TCGCACACTTGAGGTCTTCT
g6073	KAJ2949132.1	fatty acid desaturase domain-containing protein [*Phthorimaea operculella*]	0.0	95.03%	F	CTCACAATGCAGAACGAGGG
					R	CCAGGTCTCGTTCCAGAAGT
g17187	KAJ2947408.1	fatty acid desaturase domain-containing protein [*Phthorimaea operculella*]	0.0	90.82%	F	CAACAGTGCAGCCCATCTTT
					R	GCAGACAGATTGAACCGGTC
g3110	KAJ2954803.1	cuticle protein 19.8-like [*Melitaea cinxia*]	5 × 10^−63^	56.81%	F	ACGTAGCGGACTCACTTACC
					R	ATCCTGGCTGACACTACTGC
g14422	KAJ2939703.1	insect cuticle domain-containing protein [*Phthorimaea operculella*]	1 × 10^−75^	76.63%	F	CTCCCACCCCAAATACGAGT
					R	TGTTGGGGATGTTTGTGTGC
g17630	KAJ2938281.1	insect cuticle domain-containing protein [*Phthorimaea operculella*]	2 × 10^−118^	93.57%	F	CCTGGAGGCTGGTCATACAA
					R	AATGCGCGTACTTCTGTGTC
g11272	KAJ2949947.1	GMC oxidoreductase domain-containing protein [*Phthorimaea operculella*]	0.0	88.66%	F	TGTTTCCGATTTGCCAGTGG
					R	CATGTAATCGGGGTGCGATG
g15024	KAJ2942836.1	GMC oxidoreductase domain-containing protein [*Phthorimaea operculella*]	0.0	93.35%	F	ACGCAACCTAGACATCTCGT
					R	GTTGAGGACTGTTGATCGCC
g15032	KAJ2942843.1	GMC oxidoreductase domain-containing protein [*Phthorimaea operculella*]	0.0	98.10%	F	CTATGTTGGTGGCTGCGATC
					R	GTTTCATCCCCACCAGCTTC
EF1α	MZ054826	elongation factor 1 α	/	/	F	CCTGGGCACAGAGATTTCAT
					R	GATCAGCTGCTTGACACCAA
RPL28	MZ054829	ribosomal protein L28	/	/	F	TCAGACGTGCTGAACACACA
					R	GCCAGTCTTGGACAACCATT

RT-PCR, real-time polymerase chain reaction; F, forward primer; R, reverse primer.

**Table 2:** Resistance levels of *P. absoluta* to chlorantraniliprole during selection.

Generation Number	Population	Slope ± SE	LC_25_ (95% CL) (mg·L^−1^) ^a^	LC_50_ (95% CL) (mg·L^−1^)	*χ* ^2^	df	RR ^b^ (with SS)	RR (with CX-Sub_1_)
/	SS	0.465 ± 0.102	0.006 (0.000–0.035)	0.170 (0.026–0.477)	1.331	4	/	/
1st	CX-Sub_1_	1.213 ± 0.175	1.873 (0.709–3.514)	6.741 (3.609–11.180)	6.383	4	39.65	/
2nd	CX-Sub_2_	1.332 ± 0.178	2.160 (1.012–3.627)	6.933 (4.217–10.649)	1.482	4	40.78	1.03
3rd	CX-Sub_3_	0.558 ± 0.110	4.734 (1.708–9.306)	36.504 (19.817–73.235)	3.419	4	214.73	5.42
4th	CX-Sub_4_	0.983 ± 0.134	6.762 (3.206–11.597)	32.816 (19.913–56.055)	2.223	4	193.04	4.87
5th	CX-Sub_5_	0.923 ± 0.129	5.906 (2.639–10.462)	31.722 (18.793–55.760)	1.553	4	186.60	4.71
6th	CX-Sub_6_	0.791 ± 0.121	4.871 (1.828–9.392)	34.708 (19.188–67.062)	1.425	4	204.16	5.15
7th	CX-Sub_7_	0.844 ± 0.124	5.699 (2.346–10.536)	35.878 (20.458–66.788)	0.819	4	211.05	5.32
8th	CX-Sub_8_	0.947 ± 0.132	7.426 (3.455–12.895)	38.313 (22.937–67.422)	2.144	4	225.37	5.68

^a^ CL, confidence limit. ^b^ RR, resistance ratio; SS, susceptible strain; LC_50_ of resistant strain/LC_50_ of susceptible strain; SE, standard error.

**Table 3:** Development times of various life stages and fecundity for the control, CX-Sub_2_, CX-Sub_4_, and CX-Sub_8_ strains of *P. absoluta*.

Stage	Control (Mean ± SE)	CX-Sub_2_ (Mean ± SE)	CX-Sub_4_ (Mean ± SE)	CX-Sub_8_ (Mean ± SE)
Egg (days)	4.87 ± 0.05 bc	4.77 ± 0.06 c	4.98 ± 0.04 b	5.52 ± 0.06 a
Larvae 1 (days)	3.02 ± 0.07 c	2.88 ± 0.07 c	3.28 ± 0.08 b	3.59 ± 0.06 a
Larvae 2 (days)	2.81 ± 0.07 b	2.76 ± 0.09 b	3.22 ± 0.08 a	3.28 ± 0.06 a
Larvae 3 (days)	2.72 ± 0.07 c	2.80 ± 0.07 b	3.10 ± 0.08 a	3.14 ± 0.08 a
Larvae 4 (days)	2.77 ± 0.07 a	2.63 ± 0.08 a	2.52 ± 0.07 b	2.71 ± 0.09 a
Pupae (days)	7.14 ± 0.09 bc	6.94 ± 0.08 c	7.25 ± 0.12 ab	7.58 ± 0.11 a
Preadult (days)	23.26 ± 0.13 c	22.93 ± 0.17 c	24.24 ± 0.23 b	25.71 ± 0.21 a
Adult (days)	17.53 ± 0.66 b	17.54 ± 0.73 b	19.03 ± 0.61 ab	20.06 ± 0.72 a
Female adult longevity (days)	18.09 ± 0.92 b	16.80 ± 0.99 b	18.79 ± 0.78 ab	21.12 ± 1.13 a
Male adult longevity (days)	17.07 ± 0.93 a	18.27 ± 1.00 a	19.24 ± 0.93 a	18.77 ± 0.74 a
APOP (days)	2.12 ± 0.14 b	3.03 ± 0.14 a	3.11 ± 0.15 a	2.68 ± 0.19 a
TPOP (days)	25.12 ± 0.25 d	26.00 ± 0.30 c	27.34 ± 0.31 b	28.45 ± 0.32 a
Oviposition (days)	5.79 ± 0.17 a	5.23 ± 0.25 a	4.24 ± 0.19 b	4.13 ± 0.21 b
Fecundity (eggs/♀)	102.18 ± 4.68 a	71.06 ± 7.00 b	57.63 ± 4.81 bc	48.56 ± 6.30 c

SE, standard error; APOP, adult preoviposition period; TPOP, total preoviposition period. Note: Different letters represent significant differences according to the paired bootstrap test (*p* < 0.05).

**Table 4:** Life table parameters for the control, CX-Sub_2_, CX-Sub_4_, and CX-Sub_8_ strains of *P. absoluta*.

Life Table Parameters	Control (Mean ± SE)	CX-Sub_2_ (Mean ± SE)	CX-Sub_4_ (Mean ± SE)	CX-Sub_8_ (Mean ± SE)
*GRR* (offspring individual^−1^)	47.14 ± 6.40 a	32.56 ± 5.10 ab	28.37 ± 3.99 b	27.68 ± 4.50 b
*R*_0_ (offspring individual^−1^)	33.72 ± 5.05 a	24.87 ± 4.17 ab	21.90 ± 3.32 b	20.88 ± 3.60 b
*r_i_* (day^−1^)	0.13 ± 0.01 a	0.11 ± 0.01 ab	0.11 ± 0.01 b	0.10 ± 0.01 b
*T* (days)	27.82 ± 0.23 b	28.17 ± 0.29 b	28.58 ± 0.32 b	30.17 ± 0.42 a
*λ*(day^−1^)	1.14 ± 0.01 a	1.12 ± 0.01 ab	1.11 ± 0.01 b	1.10 ± 0.01 b
*Rf*	1.00	0.74	0.65	0.62

Note: Different letters represent significant differences according to the paired bootstrap test (*p* < 0.05). SE—standard error; *r_i_*—intrinsic rate of increase; *λ*—finite rate of increase; *R*_0_—net reproductive rate; *T*—mean generation time; *GRR*—gross reproductive rate; *Rf*—relative fitness rate (*Rf* = *R*_0_ of CX-Sub strain/*R*_0_ of control strain).

## Data Availability

The original contributions presented in this study are included in the article/Supplementary Material. Further inquiries can be directed to the corresponding authors.

## Supplementary Materials

The following supporting information can be downloaded at: https://www.mdpi.com/article/10.3390/insects16050524/s1, Table S1: Resistance levels of the unselected strain (CX-S) of *P. absoluta* to chlorantraniliprole during unselection; Table S2: KEGG analysis of DEG number in control and CX-Sub_8_ strains of *P. absoluta*.

## Abbreviations

The following abbreviations are used in this manuscript:

SE	Standard error
*r_i_*	Intrinsic rate of increase
*λ*	Finite rate of increase
*R*_0_	Net reproductive rate
*T*	Mean generation time
*GRR*	Gross reproductive rate
*Rf*	Relative fitness
CX-Sub_8_	Chlorantraniliprole-resistant strain continuously selected for eight generations
CX-Sub-n	Where n = 1 to n = 8 and represents the serial numbers of generations
CX-S	Unselected strains
CX-S_8_	The eighth generation of the unselected strain, the control strain
DEG	Differentially expressed gene
GO	Gene Ontology
KEGG	Kyoto Encyclopedia of Genes and Genomes
APOP	Adult preoviposition period
TPOP	Total preoviposition period
Nm/Nf	Number of males/Number of females
