# Supplementary material for: Multigeneration Sublethal Chlorantraniliprole Treatment Disrupts Nutritional Metabolism and Inhibits Growth, Development, and Reproduction of Phthorimaea absoluta
Source: Insects. 2025 May 15;16(5):524. doi: 10.3390/insects16050524 (PMC12112179; doi:10.3390/insects16050524)
Supplement: Supplementary file 1 [file insects-16-00524-s001.zip › insects-3610761-supplementary.pdf]

insects MDPI

Table S1. Resistance levels of the unselected strain (CX-S) of *P. absoluta* to chlorantraniliprole during unselection.

Population	Slope±SE	LC_{25} (95%CL) (mg L^{-1})	LC_{50} (95%CL) (mg L^{-1})	χ^2	df	RR (with SS)
SS	0.465±0.102	0.006 (0.000–0.035)	0.170 (0.026–0.477)	1.331	4	/
$CX-S_1$	1.213±0.175	1.873 (0.709–3.514)	6.741 (3.609–11.180)	6.383	4	39.65
$CX-S_2$	1.193±0.220	1.736 (0.616–3.021)	6.380 (3.885–9.566)	2.081	4	37.52
$CX-S_4$	1.549±0.233	2.236 (1.169–3.357)	6.094 (4.217–8.363)	1.274	4	35.84
$CX-S_8$	1.581±0.240	2.134 (1.115–3.195)	5.698 (3.948–7.787)	2.025	4	33.52

RR, Resistance ratio; SS, Susceptible strain; LC_{50} of resistant strain/LC_{50} of susceptible strains; SE, standard error.

Table S2. KEGG analysis of DEG number in control and $CX-Sub_8$ strains of *P. absoluta*

KEGG Pathway	ko_ID	DEgene	gene
Biosynthesis of amino acids	ko01230	30	93
Fatty acid degradation	ko00071	29	86
Glycine, serine, and threonine metabolism	ko00260	25	83
Other glycan degradation	ko00511	15	41
Carbon metabolism	ko01200	48	222
Biosynthesis of unsaturated fatty acids	ko01040	18	59
Steroid biosynthesis	ko00100	18	63
Glycerophospholipid metabolism	ko00564	15	95
Valine, leucine, and isoleucine biosynthesis	ko00290	5	7
Longevity regulating pathway - multiple species	ko04213	24	109
